# Cases Illustrating the Action of Benzoic Acid in Affections of the Urinary Organs

**Published:** 1842-05-28

**Authors:** 


### ANALECTA.

*Cases illustrating the action of Benzoic Acid in Affections of the Urinary Organs.*—[This is a new remedial agent, and in some of the troublesome diseases of these organs may be of no little service.]

Case I.—Joseph L——d, aged 77, a dissenting minister, residing in this neighborhood, became my patient some months ago, in consequence of a very painful and often difficult passage of his water, an affection under which he had laboured for years, but especially during the winter season. He has been a widower for many years, and his habits have been uniformly and strictly regular and temperate. He has been remarkably free from illness of every description previously to the present complaint. There is no perceptible disease in the prostate gland, and he has never been troubled with gouty symptoms. He has undergone a variety of treatment from his professional advisers, from which he occasionally derived partial and temporary relief. There appears to be a glairy mucous sediment in the urine, in which, on examination, some gravelly particles may be discovered. The pain, however, at this time (which was early in December last,) especially in voiding his urine, was very great, and did not yield to fomentations, anodynes, and detergent balsams, which were very judiciously resorted to. The benzoic acid, in doses of from five to ten grains, was ordered along with the balsam of copaiva, three or four times a-day, with the addition of a small quantity of the tinctura camphoræ composit., and by persisting in its use for several weeks, the urine became less turbid, and the pain and frequent desire to make water gradually left him; and, at the distance of two months from the day he commenced the benzoic acid, he described himself as free from his troublesome symptoms, and is at this time improved in his general health, in a manner which has surprised as well as gratified all who know him.

Among a considerable number of cases of patients of the Huddersfield Infirmary, to whom I have successfully administered the benzoic acid, I will select the following, all occurring within the last four months:—

Case II.—William Hastings, aged 64, a widower, and formerly a man of very intemperate habits, applied for relief for frequent pain in the loins and distress in voiding his urine, which had, especially of late, been loaded with gravelly particles, and considerable deposition of mucus. Had been treated with mercurials under an impression that his symptoms were in a great measure, if not entirely, dependant on hepatic derangement; but though relieved in many respects, yet he continued to experience frequent pain in the loins and in the region of the bladder; nor did his dysuria abate in the smallest degree for several months, though under medical treatment and in the use of the usual remedies, until he had for a considerable time taken the benzoic acid mixture, varied from time to time, according to circumstances. On the 14th of this month he visited the infirmary, with *all* his symptoms effectually relieved, and considers himself as in a state to be dismissed as cured from the institution.

Case III.—Robert Harley, aged 68, residing at Henley, was admitted an out-patient of the infirmary, January 3, with a complaint in his bladder, as he termed it, accompanied with occasional rigors and pain in the loins. He parts with his water with great difficulty, and it is usually turbid; attributes his complaint merely to cold, as he has enjoyed a good state of health, and is, from his own account, a man of temperate habits. Except occasional aperients he took no other medicine than the copaiba and benzoic acid mixture, and at his last attendance at the infirmary considered himself quite relieved in all his symptoms. This was one of the milder description of cases, arising from his age more than from any other cause.

Case IV.—John Brook, aged 57, was troubled last winter with great uneasiness in making water, for which he received relief from medicine and dietetic treatment. He was admitted an out-patient of the infirmary in the early part of this winter, with an attack of his old complaint of more than ordinary severity. The pain was very great in passing his water, which, he says, comes away in drops; says that he has been more or less troubled with this affection for three or four years, although a man of temperate habits; apparently no part is affected but the urinary organs, and his bowels have been kept regular by occasional aperients. The use of linseed-tea and barley-water causes less distress in passing water, though there is still a considerable sediment. This man took little else than the balsam of copaiba mixture, with the benzoic acid, for about six weeks, when he expressed himself as decidedly relieved, and in the enjoyment of more ease than had fallen to his lot for the last few years past.

There are some other cases of a similar description, occurring in patients at an advanced stage of life, which I find in my note-book, but differing so little either in the symptoms or the treatment, from those already described, that I should be inexcusable in occupying your pages with mere repetition. I may mention, however, that in one of the cases the use of the benzoic acid mixture was followed by a rash over a great part of the body. This, however, is a circumstance by no means peculiar to benzoic acid, as other medicines have in some instances, from some peculiarity of temperament, produced the same effect. Permit me to recommend, therefore, to your readers a further trial of the utility of this powerful acid. It is very sparingly soluble in cold water, but is soluble, without change, in alcohol. The benzoates, however, I believe, are soluble in water, and the benzoate of ammonia may, in some instances of dysuria senilis, prove more efficacious than the simple acid. The benzoic acid is a constituent of balsam of Tolu, of Peru, benzoin, storax, &c. &c. It will probably be found that, in other complaints as well as diseases of the urinary organs, its efficacy may be much greater than we have hitherto been led to expect.—***Provincial Med. and Surg. Journal.*** February 26, 1842.

